# Integrated bioinformatics, network pharmacology, and artificial intelligence to predict the mechanism of celastrol against muscle atrophy caused by colorectal cancer

**DOI:** 10.3389/fgene.2022.1012932

**Published:** 2022-11-07

**Authors:** Ming Wu, Yan Zhang

**Affiliations:** ^1^ Postgraduate Training Base in Shanghai Gongli Hospital, Ningxia Medical University, Shanghai, China; ^2^ Department of Orthopedics, Gongli Hospital of Pudong New Area, Shanghai, China

**Keywords:** bioinformatics, oncology, muscular atrophy, celastrol, miRNA–mRNA network, colorectal cancer, AlphaFold2, molecular docking

## Abstract

Muscle atrophy due to colorectal cancer severely reduces the quality of life and survival time of patients. However, the underlying causative mechanisms and therapeutic agents are not well understood. The aim of this study was to screen and identify the microRNA (miRNA)–mRNA regulatory network and therapeutic targets of celastrol in colorectal cancer causing muscle atrophy *via* blood exosomes. Datasets were downloaded from the Gene Expression Omnibus online database. Differential expression analysis was first performed using the blood exosome dataset GSE39833 from colorectal cancer and normal humans to identify differentially expressed (DE) miRNAs, and then, transcriptional enrichment analysis was performed to identify important enriched genes. Gene Ontology (GO) and Kyoto Encyclopedia of Genes and Genomes (KEGG) pathway enrichment analyses were performed by FunRich software. Using the muscle atrophy sample GSE34111, the DE mRNAs in the muscle atrophy sample were analyzed, a regulatory network map was established based on miRNA‒mRNA regulatory mechanisms, further GO and KEGG enrichment analyses were performed for the DE genes in muscle atrophy *via* Cytoscape’s ClueGO plug-in, and the network pharmacology pharmacophore analysis method was used to analyze the celastrol therapeutic targets, taking intersections to find the therapeutic targets of celastrol, using the artificial intelligence AlphaFold2 to predict the protein structures of the key targets, and finally using molecular docking to verify whether celastrol and the target proteins can be successfully docked. A total of 82 DE miRNAs were obtained, and the top 10 enriched target genes were identified. The enrichment of the 82 miRNAs showed a close correlation with muscle atrophy, and 332 DE mRNAs were found by differential expression analysis in muscle atrophy samples, among which 44 mRNA genes were involved in miRNA‒mRNA networks. The DE genes in muscle atrophy were enriched for 30 signaling pathways, and 228 target genes were annotated after pharmacophore target analysis. The *NR1D2* gene, the target of treatment, was found by taking intersections, the protein structure of this target was predicted by AlphaFold2, and the structure was successfully docked and validated using molecular docking. In our present study, colorectal cancer likely enters the muscle from blood exosomes and regulates skeletal muscle atrophy through miRNA‒mRNA regulatory network mechanisms, and celastrol treats muscle through *NR1D2* in the miRNA‒mRNA regulatory network.

## Introduction

Cachexia is a severe atrophy syndrome (Newton et al., 2020); in many chronic diseases, such as cancer, acquired immune deficiency syndrome, and tuberculosis, there is a dramatic loss of weight (Miao et al., 2017). Between 50% and 80% of cancer patients have significant symptoms characterized by fatigue (Khatib et al., 2018), loss of muscle and fat, and generalized inflammation (Miyaguti et al., 2020). Colorectal cancer is a common form of cancer (Li et al., 2020) and is also associated with cachexia (Alsolmei et al., 2019). The most striking feature of cachexia is the depletion of skeletal muscles (Vinke et al., 2020). Malignant cachexia not only reduces the quality of life of the patient (Gomes et al., 2021) but can also make radiotherapy and chemotherapy less effective and shorten the patient’s life expectancy considerably (Zhou et al., 2021).

Many factors produced by the body and cancer cells, including inflammatory cytokines, contribute to the cachexia of tumors (Sirniö et al., 2019). The recent literature has reported that the exosomes of tumor cells also play an important role in cachexia (Fan et al., 2022). Tumor exosomes are nanoscale vesicles that are secreted extracellularly by cancer cells (Chai et al., 2020) to transport substances and information in the tumor and the tumor microenvironment (Rocha et al., 2019). The exosomes (Li et al., 2021) of tumor cells and microRNAs (miRNAs) (Di et al., 2021) in exosomes play regulatory roles in malignant tumor pathogenesis. Moreover, the miRNA‒mRNA regulatory mechanism plays an important regulatory role in colorectal cancer muscle atrophy (Miao et al., 2021).

Chinese medicine has been in clinical practice in China for thousands of years (Shan et al., 2020). Numerous studies have shown that herbs have important anticancer activities (Wang et al., 2020). Celastrol, a natural product with a wide range of biological activities (Ma et al., 2022), has been documented for the treatment of colorectal cancer (Zhang W. et al., 2022) and is also useful in the treatment of cancerous malignancies (Yadav et al., 2022). Bioinformatics analysis has previously been reported for the study of muscle injury (Sun et al., 2018). Network pharmacology is a common analytical method for analyzing drug targets and can be used to find targets of drug actions using a network pharmacology approach (Jiang et al., 2019). Currently, only 20–25% (Nurminen and Hytönen, 2018) of protein structures are known due to the limitations to protein structure resolution, which makes research into drugs for disease treatment difficult. AlphaFold2 (Jumper et al., 2021) is a new artificial intelligence (AI) biocomputing technology that is able to accurately predict the structures of proteins, greatly reducing the difficulty of drug development (Schauperl and Denny, 2022).

In this study, blood exosome data from colorectal cancer were used to analyze differentially expressed (DE) miRNAs, and then, DE mRNAs were analyzed in muscle atrophy samples to model the miRNA‒mRNA regulatory network and to analyze the potential network targets of celastrol for the treatment of colorectal cancer-related muscle atrophy based on network pharmacology using AlphaFold2 and molecular docking for molecular dynamics validation. The overall goal was to provide new insights into the potential miRNA‒mRNA regulatory mechanisms of colorectal cancer muscle atrophy and the targets of celastrol therapy and explore the role of the AI AlphaFold2 in drug development.

## Materials and methods

### Data collection

First, the keywords “colorectal cancer” and “muscular atrophy” were retrieved from the Gene Expression Omnibus (GEO) database (Barrett et al., 2013), and the GSE39833 (Ogata-Kawata et al., 2014) and GSE34111 (Gallagher et al., 2012) microarray datasets were selected as research objects for download. GSE39833 is a microarray dataset of miRNAs on the GPL14767 platform, which includes exosome samples from 11 healthy controls and 88 colorectal cancer patients. GSE34111 is a microarray chip dataset for muscular dystrophy disease, and the platform is GPL570. We selected six healthy samples from the control group and 12 samples from the muscular dystrophy group for data analysis.

### Analysis of differentially expressed miRNAs

The samples were grouped according to the two groups of the GSE39833 dataset and analyzed by the “limma” package (Ritchie et al., 2015) in R (4.1.0) software. |Log2(fold change)| > 1 and *p* < 0.05 were used as screening conditions to identify significant DE miRNAs.

### Transcription factors of DE miRNAs and enrichment analysis

According to the functional enrichment and interactive network analysis tool FunRich (3.1.3), TFs enriched with DE miRNAs were determined, and the top 10 significant TFs were graphically displayed. Gene Ontology (GO) terms were annotated and mapped by FunRich software, including biological processes, cell composition, and molecular function, and Kyoto Encyclopedia of Genes and Genomes (KEGG) pathways were identified.

### Analysis of differentially expressed mRNAs

In the GSE39833 dataset, we selected samples from six healthy controls and 12 patients with muscle atrophy in the R software package “limma” for differential expression analysis, with the filtering conditions |log2 FC (fold change)| > 0.5 and *p* < 0.05. DE mRNAs with significant expression were screened out.

### DE miRNA target gene prediction and construction of the miRNA‒mRNA gene regulatory network

The miRNA target genes were predicted within three databases: miRTarBase, miRDB, and TargetScan. Subsequently, we constructed miRNA‒mRNA gene regulatory networks for the screened DE miRNAs with DE mRNAs based on miRNA‒mRNA regulatory mechanisms. Cytoscape (version 3.9.0) was used to visualize miRNA‒mRNA gene regulatory networks.

### Enrichment analysis of differentially expressed mRNAs

DE mRNAs were imported into Cytoscape’s ClueGO plug-in (Bindea et al., 2009), and “*Homo sapiens*” was selected for GO enrichment analysis, including the biological process, cell composition, and molecular function, as well as KEGG signaling pathway enrichment analysis, and the map was drawn.

### Construction of a network target for celastrol

PharmMapper is an online platform for pharmacophore matching and potential identification of targets. The PharmMapper (http://www.lilab-ecust.cn/pharmmapper/) database was used to select the top 600 ranked targets for pharmacophore target prediction. The names of the analyzed target genes were annotated in the UniProt (https://www.uniprot.org/) database by selecting the species “*Homo sapiens*,” and the network was constructed in Cytoscape based on the drug–target relationships.

### AlphaFold2 prediction of the protein structure

AlphaFold2 is an artificial intelligence program that predicts protein structure online. We selected the target gene *NR1D2* for celastrol, found the corresponding human species protein number in the UniProt database, performed an online structure prediction search in AlphaFold2 (https://alphafold.ebi.ac.uk/search), and downloaded a PDB format file of the corresponding NR1D2 protein structure, a display map of the protein structure, the protein sequence map used, and the predicted confidence map.

### Molecular docking

The 2D structure of the small-molecule ligand celastrol was downloaded from the PubChem database. NR1D2 was selected as the protein receptor, and the PDB file predicted by the AI AlphaFold2 was used. AutoDockTools was used to read the protein receptor file, which was converted into the PDBQT format after hydrotreating ion modification. This structure was then converted to a 2D structure to draw active pockets. Finally, AutoDock Vina was used for molecular docking, and the model with the lowest free energy was selected for visualization.

### Statistical analysis

R (4.1.0) was used for bioinformatics analysis, and the R package was used for statistical analysis. *p* < 0.05 was considered statistically significant.

## Results

### Identification of 82 DE miRNAs

**FIGURE 1:**
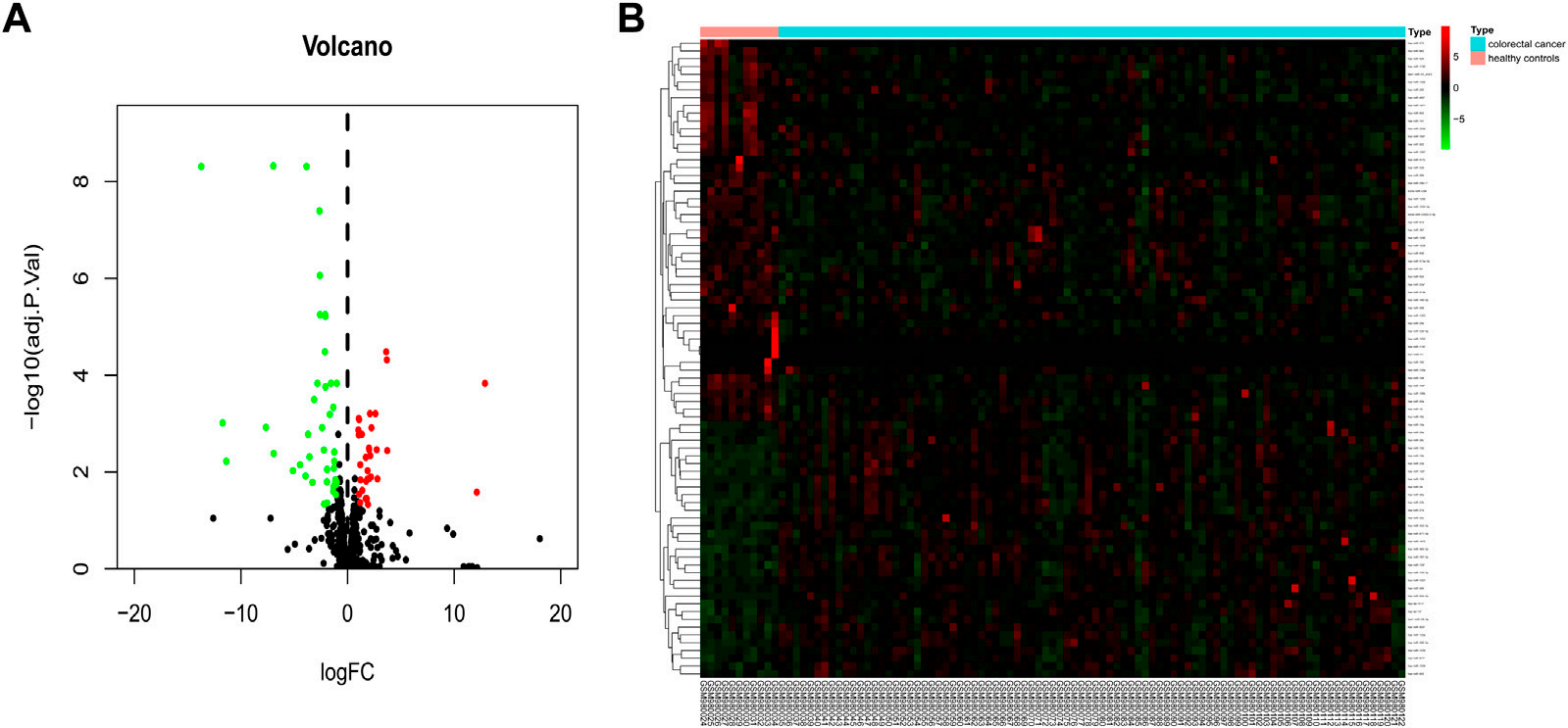
Identification of 82 DE miRNAs. Identification of differentially expressed exosomal miRNAs in colorectal cancer and healthy human blood. **(A)** Volcano map of genome-wide DE miRNAs. **(B)** Heatmap showing the total upregulated and downregulated miRNAs screened. Green and red represent downregulated and upregulated miRNAs, respectively. miRNAs, microRNAs.

To identify blood exosomal DE miRNAs caused by colorectal cancer, the GSE39833 dataset was first analyzed for differences, and under the filtering conditions of |logFC| > 1 and *p* < 0.05, a total of 82 DE miRNAs were obtained in the colorectal cancer group compared to the healthy group (Figure 1A), with 33 upregulated genes and 49 downregulated genes (Figure 1B).

### Transcription factor enrichment of differentially expressed miRNAs

**FIGURE 2:**
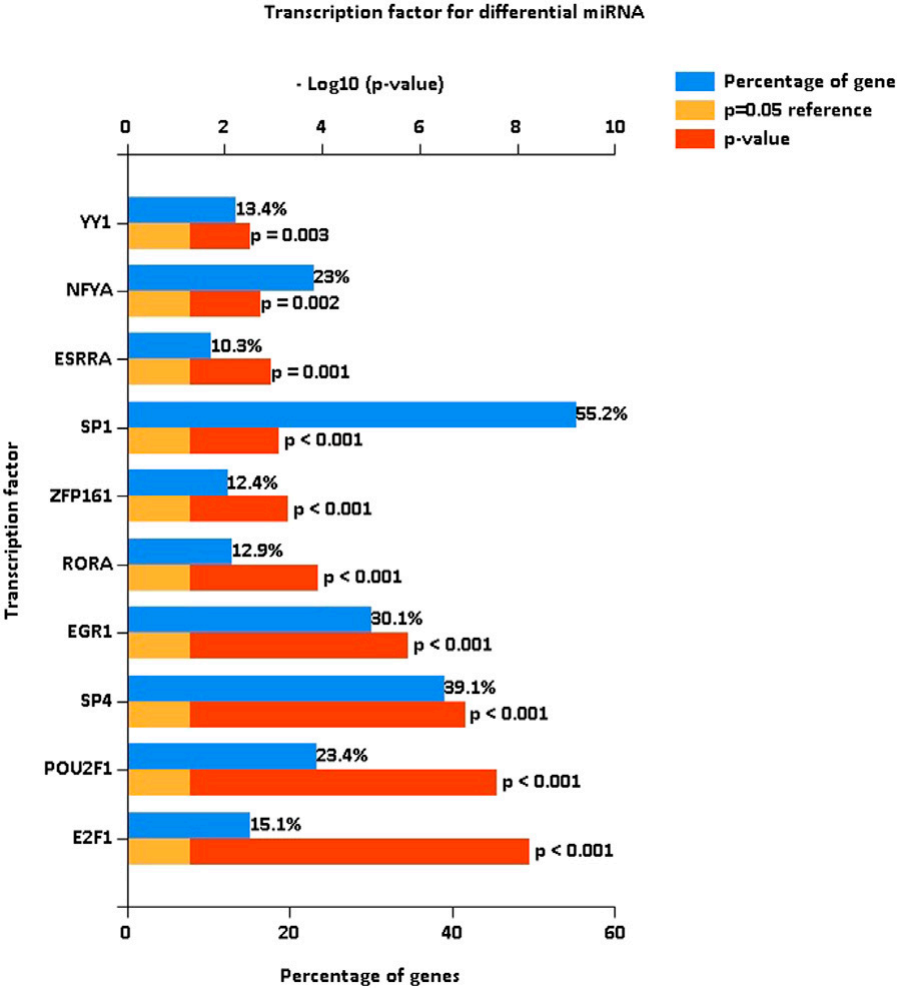
Transcription factor enrichment of differentially expressed miRNAs. Enrichment analysis of transcription factors, with the top 10 genes enriched for display. The blue bars are the proportion enriched, and the red bars indicate the *p*-values of the enrichment proportions.

After the identification of DE miRNAs, the important TFs enriched by DE miRNAs were further identified by TF enrichment analysis, and the top 10 genes (*YY1*, *NFYA*, *ESRRA*, *SP1*, *ZFP161*, *RORA*, *EGR1*, *SP4*, *POU2F1*, and *E2F1*) were graphically displayed (Figure 2).

### Enrichment analysis of differentially expressed miRNAs

**FIGURE 3:**
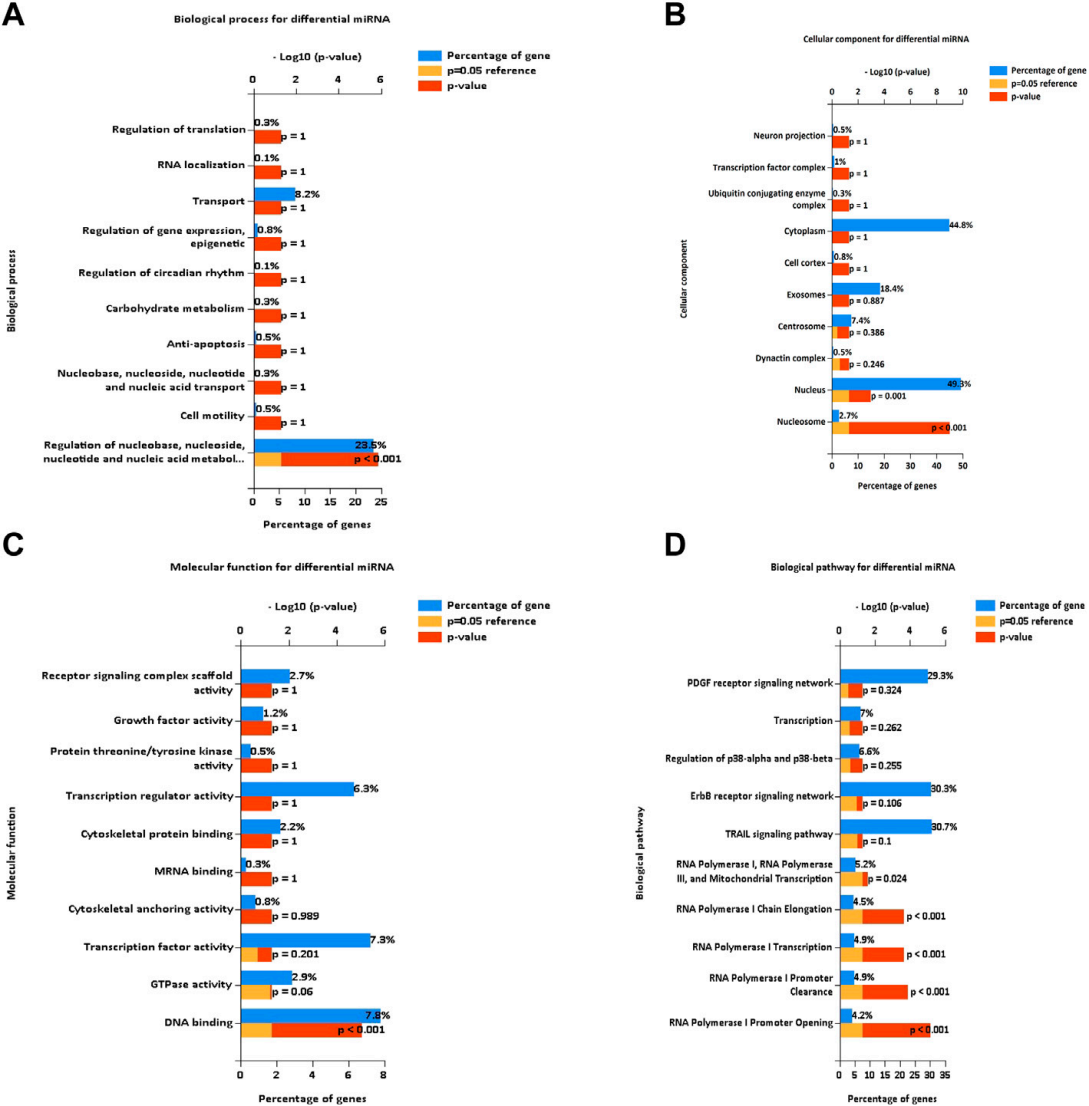
Enrichment analysis of differentially expressed miRNAs. GO and KEGG enrichment analyses of DE miRNAs. **(A)** Bar diagram of BP. **(B)** Histogram of CC. **(C)** Histogram of MF. **(D)** Bar diagram of KEGG. GO, Gene Ontology; BP, biological process; CC, cellular component; MF, molecular function; KEGG, Kyoto Encyclopedia of Genes and Genomes.

To determine the functions and signaling pathways of DE miRNA enrichment, FunRich software was used for GO and KEGG enrichment analyses of DE miRNAs. Biological process (BP) (Figure 3A) enrichment included regulation of circadian rhythm and lipid metabolism, cellular component (CC) (Figure 3B) enrichment included nucleosome and exosomes, and molecular function (MF) (Figure 3C) enrichment included transcription factor activity and mRNA binding. KEGG (Figure 3D) enrichment included RNA polymerase I promoter opening and the circadian rhythm pathway.

### Identification of differentially expressed mRNAs in muscular atrophy

**FIGURE 4:**
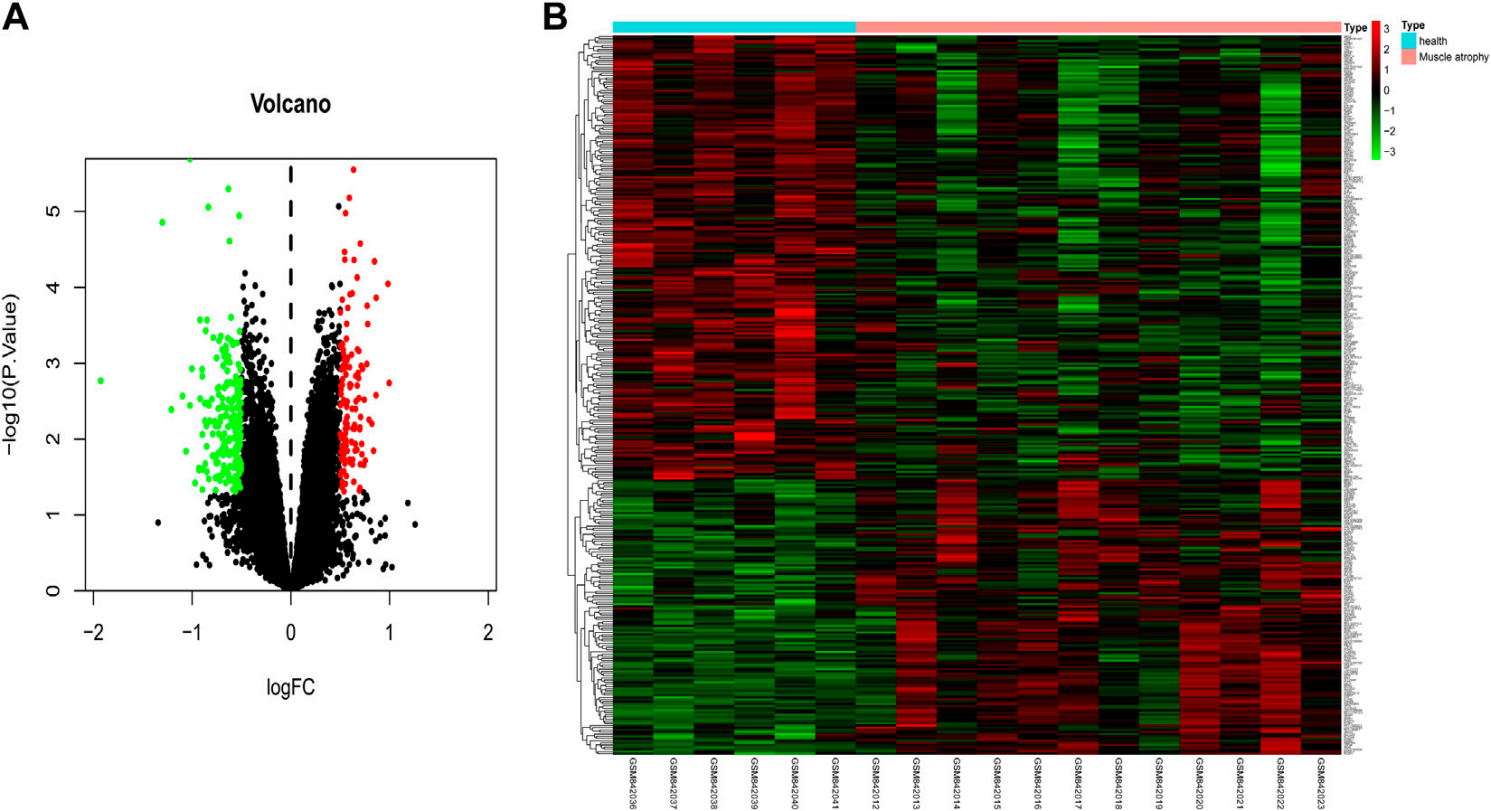
Identification of differentially expressed mRNAs in muscular atrophy. Identification of DE mRNAs in muscle samples from patients with muscular dystrophy and healthy individuals. **(A)** Volcano map of genome-wide DE mRNAs. **(B)** Heatmap showing all of the upregulated and downregulated mRNAs screened. Green and red represent downregulated and upregulated mRNAs, respectively.

To identify DE mRNAs in the muscle atrophy samples, differential expression analysis was performed on the GSE34111 dataset using the limma package of R software, and a total of 332 DE mRNAs were obtained in the muscle atrophy group compared to the healthy group under a filter of |logFC| > 0.5 and *p* < 0.05 (Figure 4A), of which 127 were upregulated and 205 were downregulated. The results are displayed in a heatmap in Figure 4B.

### Prediction of target genes for differentially expressed miRNAs and construction of miRNA‒mRNA gene regulatory networks

**FIGURE 5:**
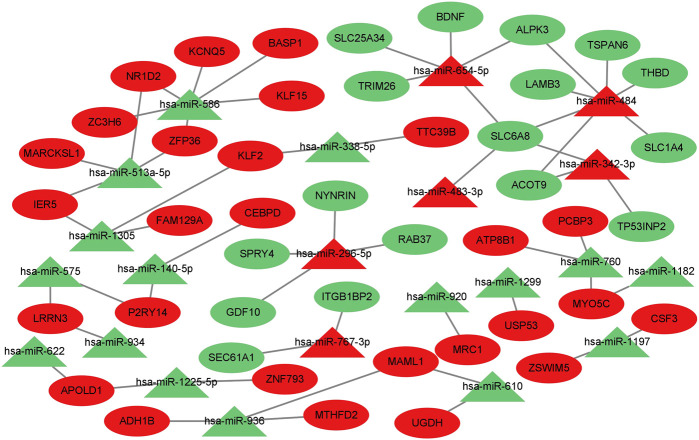
Prediction of target genes for differentially expressed miRNAs and construction of a miRNA‒mRNA gene regulatory network. miRNA‒mRNA gene regulatory network. miRNAs are shown as triangles, and target genes are shown as ovals. Red indicates upregulated genes, and green indicates downregulated genes.

To further identify the miRNA‒mRNA gene regulatory network of colorectal cancer that regulates muscle atrophy *via* exosomes, we predicted the target genes of DE miRNAs using three databases and subsequently intersected the predicted target genes with DE mRNAs to obtain intersecting genes. Furthermore, based on the mechanism of miRNA‒mRNA regulation, a miRNA‒mRNA network was constructed using Cytoscape (Figure 5), in which there were 44 mRNAs and 22 miRNAs (hsa-miR-484, hsa-miR-654–5p, hsa-miR-610, hsa-miR-1305, hsa-miR-575, hsa-miR-760, hsa-miR-934, hsa-miR-936, hsa-miR-342–3p, hsa-miR-140–5p, hsa-miR-338–5p, hsa-miR-1197, hsa-miR-767–3p, hsa-miR-586, hsa-miR-1225–5p, hsa-miR-296–5p, hsa-miR-513a-5p, hsa-miR-622, hsa-miR-1299, hsa-miR-1182, hsa-miR-483–3p, and hsa-miR-920).

### Enrichment analysis of differentially expressed mRNAs in muscular atrophy

**FIGURE 6:**
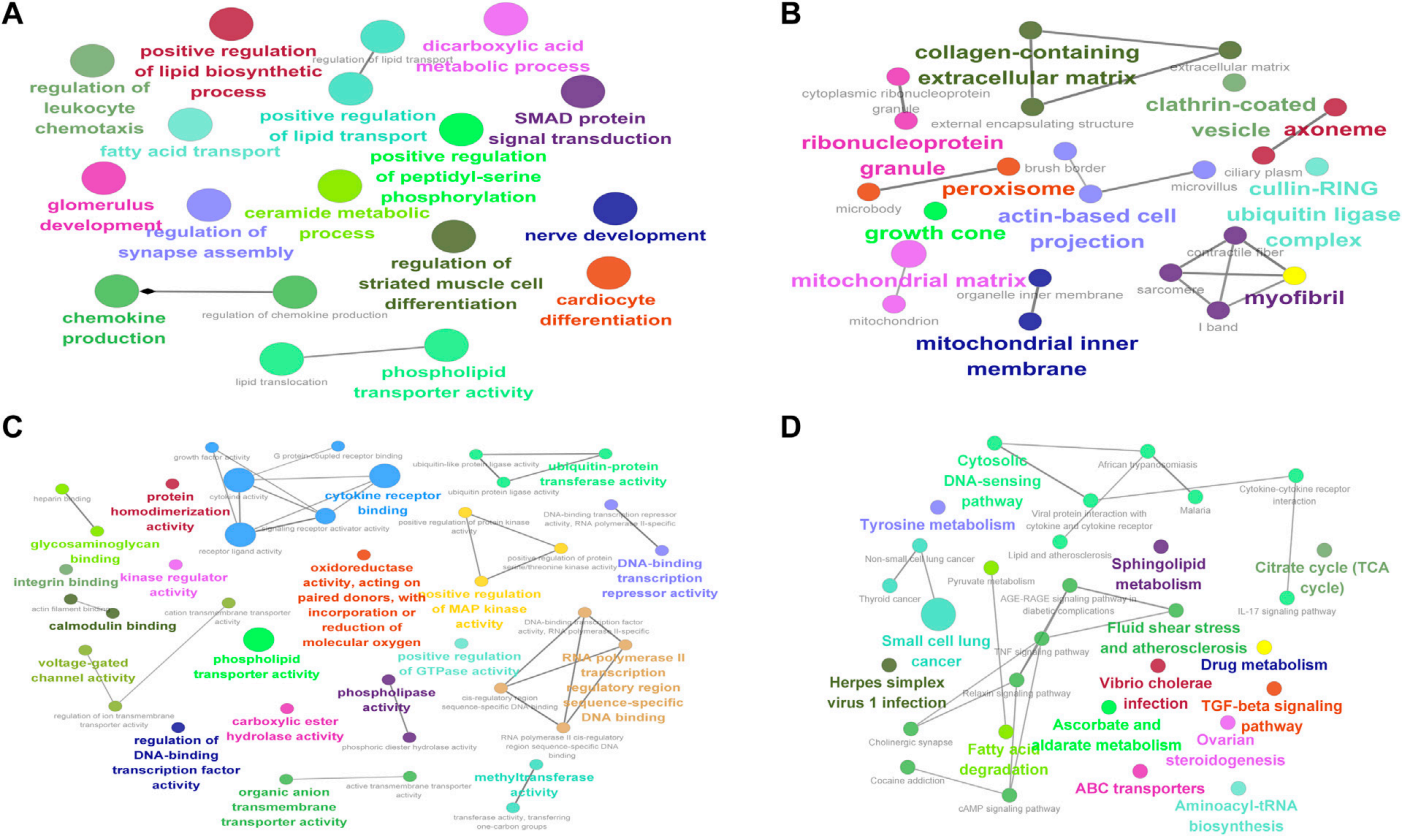
Enrichment analysis of differentially expressed mRNAs in muscular atrophy. GO and KEGG enrichment analyses of DE mRNAs. **(A)** Circle diagram of BP. **(B)** Circle diagram of CC. **(C)** Circle diagram of MF. **(D)** Circle diagram of KEGG. The color represents the *p*-value; the darker the color is, the smaller the *p*-value is. The size of the circle represents the number of genes. BP, biological process; CC, cellular component; MF, molecular function; KEGG, Kyoto Encyclopedia of Genes and Genomes.

To determine the GO and KEGG enrichment programs of DE mRNAs, the ClueGO plug-in was used for the enrichment analysis of DE mRNAs. BP (Figure 6A) enrichment included regulation of lipid transport and positive regulation of lipid biosynthetic processes, CC (Figure 6B) enrichment included proteinase and proteinase sarcomeres, and MF (Figure 6C) enrichment included actin filament binding and phospholipid transporter activity (Figure 6D). KEGG enrichment included pyruvate metabolism and the TGF-beta signaling pathway.

### Construction of the celastrol pharmacophore target network

**FIGURE 7:**
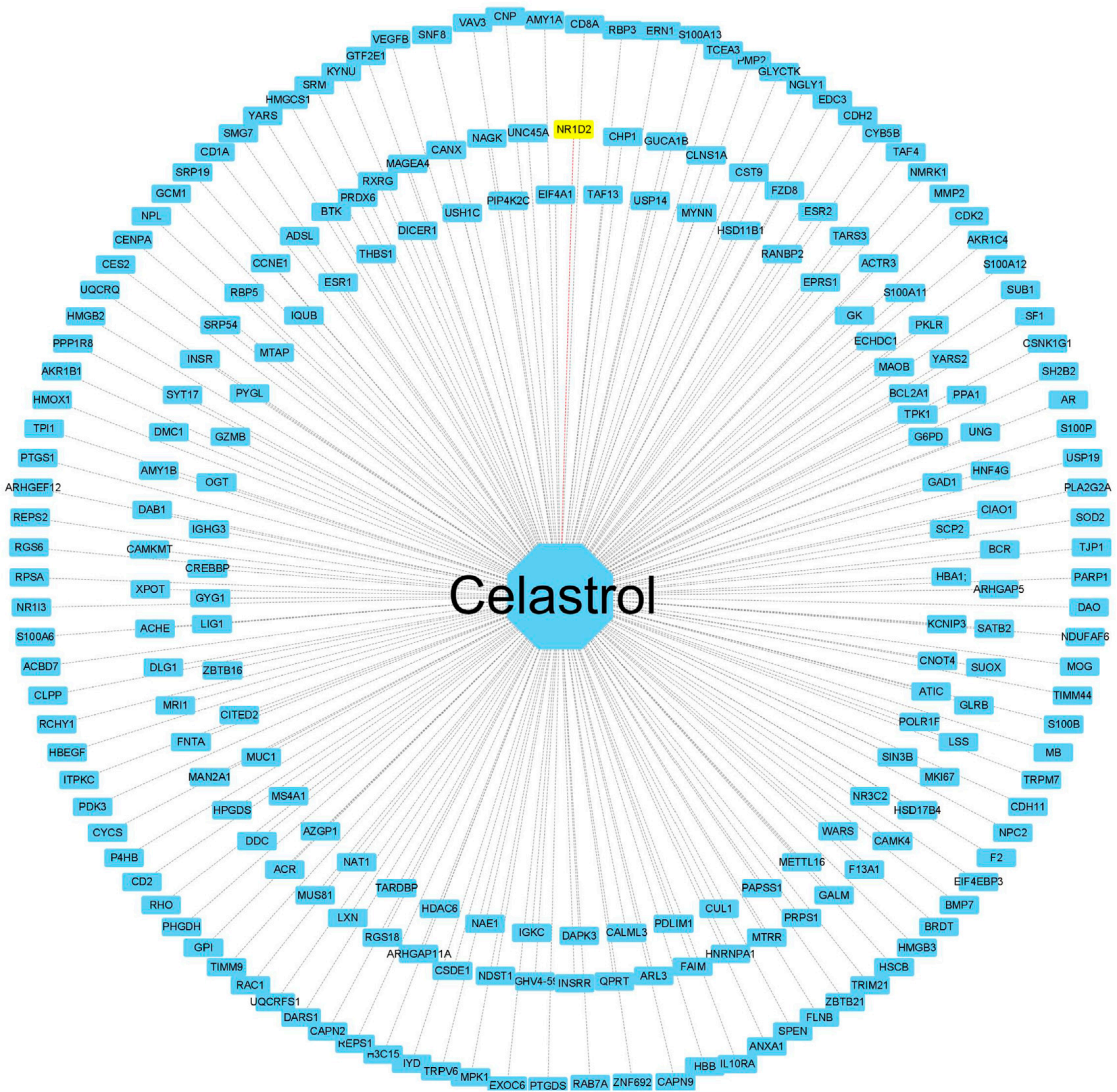
Construction of the celastrol pharmacophore target network. The “drug–pharmacophore target” network. The drug ryanodine is in the middle, and the network dots are the annotated successful pharmacophore gene targets (yellow indicates the drug and the corresponding disease target gene). The middle line is the target relationship linkage.

To create a target network map of celastrol, pharmacophore target genes were predicted by the PharmMapper database, and 228 genes were successfully annotated by the UniProt annotation database. The mRNAs of the annotated pharmacophore genes were intersected with those of the miRNA‒mRNA gene network to obtain NR1D2, the drug therapeutic target gene celastrol. The annotated genes were then imported into Cytoscape software to complete the network construction of celastrol and target genes (Figure 7).

### Artificial intelligence AlphaFold2 predicts the NR1D2 protein structure

**FIGURE 8:**
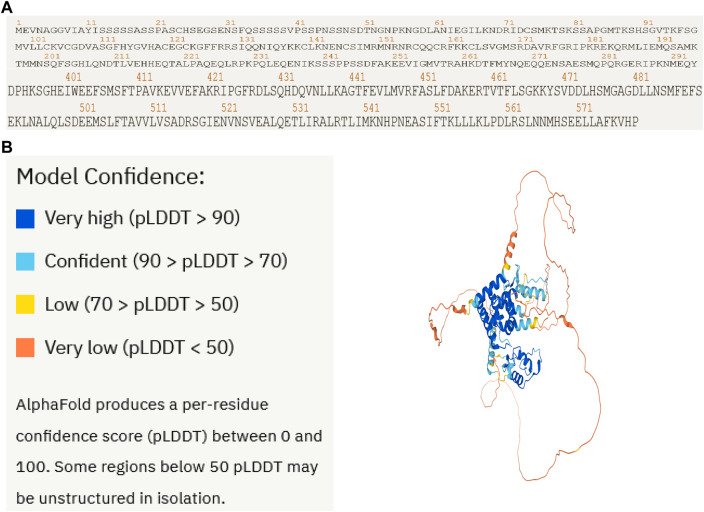
Artificial intelligence AlphaFold2 predicts the NR1D2 protein structure. Protein structure of the therapeutic target gene *NR1D2* predicted by the AI AlphaFold2. **(A)** Coding sequence of NR1D2. **(B)** Protein structure predicted by the AI AlphaFold2.

To confirm the protein structure of NR1D2, the human protein number Q14995 of the gene was first found through the UniProt online database and then predicted by the AI AlphaFold2, which automatically identifies the amino acid sequence of the protein based on the protein code (Figure 8A) and calculates the sequence to generate the spatial structure of the protein (Figure 8B).

### Celastrol docks with NR1D2 molecules

**FIGURE 9:**
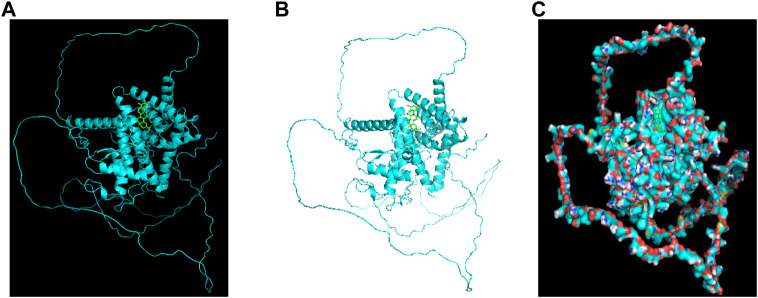
Celastrol docks with NR1D2 molecules. Molecular docking of the small-molecule ligand celastrol to the protein receptor of the target gene *NR1D2*. **(A,B)** Lowest free energy molecular docking. **(C)** Molecular docking of the lowest free energy protein surface.

To further validate the network pharmacological predictions, the drug target celastrol for the treatment of colorectal cancer muscular atrophy was elaborated. We performed molecular docking validation of the protein structure of the target gene *NR1D2*, with center_x = −2.472, center_y = 1.935, and center_z = −10.225 as the active center for docking and selected the lowest free energy of −8.4 for demonstration (Figures 9A–C).

## Discussion

Cancer can lead to wasting and muscle atrophy (Chang et al., 2021). When complications of severe muscle atrophy occur with cancer cachexia, it can produce high mortality rates, especially in advanced tumors; moreover, no effective treatment drugs are currently available (Dasgupta et al., 2020).

The proliferation of tumor cells results in the secretion of a large number of vesicles, including exosomes, which may play a broad regulatory role in the development of cancer cachexia (Rao et al., 2020). Celastrol has been reported in the past literature to have anticancer effects on colon cancer (Moreira et al., 2022), inhibit colorectal cancer by inducing apoptosis in colorectal cancer cells (Zhang H. et al., 2022), have significant inhibitory effects on colorectal cancer (Wang S. et al., 2019), have good therapeutic effects on muscle atrophy (Kitahata et al., 2022), resist muscle atrophy (Gwag et al., 2013), and induce muscle fiber preservation (Gwag et al., 2015). In our present study, we used bioinformatics to analyze the potential miRNA‒mRNA regulatory network of colorectal cancer *via* exosomes to regulate muscle atrophy through network pharmacology and the AI AlphaFold2 in an attempt to elucidate the possible regulatory mechanisms of celastrol *via* the miRNA‒mRNA regulatory network on colorectal cancerous muscle atrophy.

The blood exosome dataset GSE39833 from colorectal cancer and normal humans was first downloaded through the GEO database. Differential expression analysis by the limma package yielded 82 DE miRNAs. Of these miRNAs, 33 were upregulated and 49 were downregulated. Subsequently, 82 DE miRNAs were analyzed for TF enrichment and then for GO and KEGG enrichment. The results of GO enrichment showed regulation of circadian rhythm, lipid metabolism, nucleosome, exosomes, and transcription factor activity. KEGG enrichment results showed the RNA polymerase I promoter opening and circadian rhythm pathway. Then, 332 DE mRNAs were obtained by differential expression analysis of the muscle atrophy sample GSE34111. mRNA targets were obtained by online database analysis of DE miRNAs, and a regulatory network model diagram was established according to the principle of miRNA‒mRNA regulation, with 22 miRNAs and 44 mRNAs in the network. Of these, CEBPD has been reported to be a biomarker for amyotrophic lateral sclerosis (Sun et al., 2022). *BDNF* can improve muscle atrophy by promoting nerve regeneration (Yongguang et al., 2022) and is a target for skeletal muscle inflammation (Aby et al., 2021). *TP53INP2* is a key regulator of skeletal muscle (Sala and Zorzano, 2015) and regulates muscle atrophy due to cancer cachexia by activating autophagy (Penna et al., 2019). Piezo1/KLF15/IL-6 axis-induced muscle atrophy (Hirata et al., 2022) is an important regulatory pathway for muscle atrophy in mice (Jagasia and Wagner, 2022). *SLC6A8* knockdown results in a decrease in muscle mass (Duran-Trio et al., 2022). *KLF2* regulates skeletal muscle injury and regeneration (Manoharan et al., 2019). *ALPK3* is associated with cardiomyopathy (Jorholt et al., 2020). *MAML1* is essential for normal muscle production (Shen et al., 2006). *ITGB1BP2* is likely to be involved in myotube fusion (Jaka et al., 2017). *SLC1A4* is involved in the regulation of myocytes (Frese et al., 2015). *Spry1* and *Spry4* regulate the human aortic smooth muscle cell phenotype through differential Akt/FoxO/cardiomyosin signaling (Yang et al., 2013). *ZFP36* targets myogenic transcripts and may play a role in adult muscle stem cells (Bye-A-Jee et al., 2018). *BASP1* plays an important role in vascular smooth muscle (Santiago et al., 2021). *KCNQ5* is involved in smooth muscle contractility regulation (Wang L. et al., 2019). There is an association between *NR1D2* clock gene expression and mitochondrial quality control, while impaired oxidative capacity and mitochondrial function contribute to Duchenne muscular dystrophy (Hardee et al., 2021). *MRC1* is a biomarker for macrophage relief of cisplatin-induced sarcopenia (Hong et al., 2021). The miRNA‒mRNA network that we make regulates other genes in the network, such as *TRIM26*, *UGDH*, *LRRN3*, *ATP8B1*, *ACOT9*, *LAMB3*, *TTC39B*, *CSF3*, *SLC25A34*, *ZC3H6*, *MTHFD2*, *APOLD1*, *PCBP3*, *FAM129A*, *RAB37*, *ZNF793*, *SEC61A1*, *IER5*, *TSPAN6*, *P2RY14*, *THBD*, *ADH1B*, USP53, *MYO5C*, *MARCKSL1*, *ZSWIM5*, and *NYNRIN*. The regulation of these genes needs further research.

Subsequently, we conducted GO and KEGG enrichment analyses on DE mRNAs. GO enrichment mainly included the regulation of lipid transport, positive regulation of the lipid biosynthetic process, mitochondrion and sarcomere, actin filament binding, IL-17 signaling pathway, and phospholipid transporter activity. KEGG enriched 30 signaling pathways, among which the important signaling pathways included the citrate cycle (TCA cycle), ascorbate and aldarate metabolism, drug metabolism, TGF-beta signaling pathway, tyrosine metabolism, sphingolipid metabolism, fatty acid degradation, pyruvate metabolism, TNF signaling pathway, relaxin signaling pathway, and fluid shear stress and atherosclerosis. The citrate cycle (TCA cycle), pyruvate metabolism, and fatty acid degradation have also been predicted to be important signaling pathways in muscle atrophy in spinal cord injury (Huang et al., 2021). The TGF-beta signaling pathway has been previously reported to play an important regulatory role in muscle atrophy (Yoshihara et al., 2022) and is an important signaling pathway in the regulation of muscle atrophy (Liu et al., 2014). Sphingolipid metabolism also affects muscular atrophy but is rarely considered (De Larichaudy et al., 2012). Ascorbate and aldarate metabolism, drug metabolism, tyrosine metabolism, pyruvate metabolism, the TNF signaling pathway, the relaxin signaling pathway, the IL-17 signaling pathway, and fluid shear stress and atherosclerosis need further study.

The therapeutic targets of celastrol were analyzed by pharmacophore target analysis of network pharmacology, and 228 genes were successfully annotated and then intersected with mRNAs of the miRNA‒mRNA regulatory network to find the disease target gene *NR1D2* for drug treatment. In GeneCards, NR1D2 is presented as a transcriptional repressor that coordinates circadian rhythms and metabolic pathways in a hemoglobin-dependent manner. NR1D2 is a component of complex transcriptional machinery that controls circadian rhythms and forms a key pathway in the biological clock by directly repressing the expression of the core clock components ARNTL/BMAL1 and CLOCK. It also regulates genes involved in metabolic functions, including lipid metabolism and inflammatory responses. In the previous literature, NR1D2 was shown to regulate mitochondrial function by regulating the circadian rhythm, and impaired mitochondrial function leads to Duchenne muscular dystrophy. Many important signaling pathways in the KEGG enrichment of DE mRNAs in muscle atrophy are also associated with hypo, including lipid metabolism and inflammatory responses.

We then used the AI AlphaFold2 on the protein structure of NR1D2 to predict the human protein structure of the gene, successfully molecularly docking it to celastrol.

AlphaFold2 is a revolutionary change in the field of biology, demonstrating the power of AI, which is changing the way drugs are developed, greatly accelerating the development of new drugs, and helping millions of patients around the world who are unable to develop drugs because of unknown protein structures. AlphaFold2 will be a powerful AI weapon in the fight against various diseases. We offer a new strategy for drug development by combining bioinformatics, network pharmacology, and artificial intelligence.

## Conclusion

In this study, we identified a potential miRNA‒mRNA regulatory network of colorectal cancer regulating muscle atrophy through exosomes in a bioinformatics approach. There are 22 miRNAs and 44 mRNAs in this network. Molecular docking validation by the AI AlphaFold2 predicted the structure of the NR1D2 protein and revealed the molecular target of celastrol for the treatment of colorectal muscular atrophy. AlphaFold2 will help develop potential therapeutic drugs for more diseases.

## Data Availability

Publicly available datasets were analyzed in this study. The names of the repository/repositories and accession number(s) can be found in the article/supplementary material.

## Abbreviations

AI, artificial intelligence; GEO, Gene Expression Omnibus; BP, biological process; CC, cellular component; MF, molecular function; KEGG, Kyoto Encyclopedia of Genes and Genomes; DE, differentially expressed; GO, Gene Ontology; LogFC, log two-fold change; TF, transcription factor.
